# Effects of coupled application of magnetoelectric activated water and amendments on photosynthetic physiological characteristics and yield of maize in arid regions

**DOI:** 10.3389/fpls.2024.1497806

**Published:** 2025-01-16

**Authors:** Qingyuan Lei, Wanghai Tao, Fan Yang, Jianqi Liu, Zixuan Xi, Quanjiu Wang, Mingjiang Deng

**Affiliations:** State Key Laboratory of Eco-hydraulics in Northwest Arid Region, Xi’an University of Technology, Xi’an, China

**Keywords:** light response model, photosynthetic traits, quality, spring maize, yield

## Abstract

Seeking effective improvement agent control measures to enhance the photosynthetic physiological traits and yield levels of spring maize is crucial for efficient green agriculture in arid regions. Therefore, this study was conducted to clarify the effects of coupling improvement agents under magnetoelectric activated water irrigation conditions on the photosynthetic physiological traits, grain nutrients, and yield of spring maize in the arid region of northwest China. Field experiments were set up with three concentrations of growth regulators: 400 times (G1), 500 times (G2), and 600 times (G3), and three amounts of *Bacillus subtilis*: 15 kg/ha (R1), 45 kg/ha (R2), and 75 kg/ha (R3), along with a control group CK, making a total of 10 treatments applied in the field experiment. The results indicate that under magnetoelectric activated water irrigation, coupling improvement agents significantly enhance the photosynthetic traits, grain nutrients, and yield of spring maize in arid areas. With the coupling of improvement agents, the rectangular hyperbola correction model showed a good fit for the light response curve (*R^2^* >0.992). *P_nmax_* was significantly increased (7.37%~37.46%) and was highly correlated with yield (*P<0.01*). The entropy-weight TOPSIS comprehensive evaluation analysis found that the G2R2 treatment is the optimal improvement agent coupling measure for efficient production of spring maize in arid regions. This treatment yielded 12.68 t/ha and increased 100-kernel weight, grains per spike, and soluble sugar content by 21.3%, 8.22%, and 63.81%, respectively, representing the best balance of quality and high yield. The results of this study provide theoretical references and technical support for the high-quality and efficient production of spring maize in China’s arid regions.

## 1 Introduction

In an era of rapid global economic development, increasing social pressure, and enhancing agricultural productivity is crucial for promoting regional economic growth and social stability, especially in the face of continuous population growth, global warming, and other destabilizing factors (Leghari et al., 2021). In arid regions where soil salinization is widespread, the high-quality development of modern ecological agriculture faces numerous challenges, including freshwater scarcity and salt-alkali stress, leading to adverse outcomes such as land degradation, reduced plant diversity, and even crop yield decline (Zhang et al., 2024). Magnetoelectric activated water technology, which treats irrigation water with a magnetic field before agricultural use, can effectively mitigate these negative impacts by altering water quality. Additionally, magnetoelectric activated water significantly improves soil salt leaching and water use efficiency, demonstrating positive effects on enhancing soil water retention, promoting crop growth, and increasing yields (Dobránszki, 2023). Xinjiang, located in the typical arid region of northwest China, faces significant constraints on agricultural development due to arid climates, water shortages, soil salinization, and high temperatures with intense evaporation (Zhang et al., 2017a). Effectively coordinating agricultural production and land salinization in arid areas is crucial for ensuring high-quality and efficient regional agricultural production, which is strategically important for global food security in arid regions (Lin et al., 2024b).

In the context of resource scarcity, pursuing effective, integrated improvement measures is essential to enhancing crop productivity in arid regions. Maize (*Zea mays L.*), as a major crop in Xinjiang (Geng et al., 2020), has advantages such as high yield and strong adaptability (Gong et al., 2015). Additionally, maize is one of the world’s three major food crops. It can be used directly for food, and livestock feed production (Lunduka et al., 2019), and is a primary source for many industrial products (Zhang et al., 2022). The extensive application value of maize makes it the most widely planted and economically productive cereal crop globally (Wang et al., 2019b). Therefore, the safe and efficient production of maize profoundly impacts the stable development of the global agricultural economy. Currently, traditional measures to increase maize yield include increasing fertilizer application and irrigation (Cui et al., 2024), raising planting density (Shao et al., 2024), implementing intercropping and relay planting (Huang et al., 2024), and adopting conservation tillage (Adil et al., 2024). However, achieving new goals for modern, high-quality, and efficient agricultural production imposes higher demands on maize yield in arid regions. New salt-alkali land improvement measures that have emerged include engineering, biological, chemical, and biological modifications (Yang et al., 2022; Cheng et al., 2023; Wang et al., 2023a).

Currently, building on traditional improvement measures, the application of bio-amendments and other types of chemical amendments in agriculture has become a key focus in modern agricultural research. Applying appropriate soil amendments to saline-alkali land or foliar spraying of growth regulators as part of new integrated control technologies is an effective approach for improving saline-alkali soil and enhancing crop quality and productivity (Haider et al., 2024), It offers strong advantages in environmental sustainability, making it widely applicable. These methods offer strong advantages in environmental sustainability, making them widely applicable. *Bacillus subtilis* is a type of rhizosphere growth-promoting bacteria (PGPR), which are soil bacteria living on the surface of host plant roots (Gao et al., 2022). These beneficial bacteria promote root extension, suppress pests and diseases, and enhance crop growth and development through various mechanisms. Additionally, PGPR often helps roots resist biotic and abiotic stressors. Their proliferation provides benefits to the host and effectively enhances crop resistance, making them widely used in saline-alkali soil microbial improvement (Khademian et al., 2019; Bai et al., 2023). Plant growth regulators (PGRs) are chemicals that regulate and control plant growth and development, similar to plant hormones like gibberellins, ethylene, auxins, and abscisic acid.

Different types of PGRs can either promote or inhibit plant growth. These regulators come in various forms and are typically applied via foliar spray or drip irrigation for optimal crop absorption (Melini et al., 2023). As an emerging technology, PGRs are widely used in crop production for significantly enhancing growth, quality, and yield. Numerous studies show that foliar-applied PGRs significantly enhance growth and yield across various crop types (Ma et al., 2022). found that applying poly-γ-glutamic acid (γ-PGA) to spring maize in arid regions enhanced drought resistance by promoting photosynthesis, highlighting the potential of PGRs in improving crop resilience in dry environments. Recent studies have shown that *Bacillus subtilis* plays a significant role in crop growth and physiological regulation. Field experiments in Greece examined various PGRs and application methods, finding significant increases in maize’s net photosynthesis and transpiration rates, with total solids increasing by over 90% under *Bacillus subtilis* treatment (Efthimiadou et al., 2020). In conclusion, previous studies have significantly advanced the understanding of the interactions between plant growth-promoting bacteria, soil, and crops (Brown and Saa, 2015). However, comprehensive studies on the soil-plant-amendment system are needed to fully explore the regulatory potential of PGPRs and PGRs.

Studying the synergistic effects of PGPR and growth regulators to optimize photosynthetic efficiency is an effective way to enhance crop growth and increase yield. Light is the fundamental determinant of photosynthesis, a key process in plant growth, development, and reproduction. Studies show that maize yield is often determined by the performance of the photosynthetic system (Wang et al., 2023b), which is also a crucial factor in the carbon-water cycle of the terrestrial ecosystem (Wu et al., 2019). The light response curve effectively evaluates a plant’s ability to utilize light and adapt to its environment. Accurately analyzing this curve and its parameters is vital for studying the response of photosynthetic processes to environmental changes (Begam et al., 2024). The mechanisms behind the coupling of amendments for improving maize quality and efficiency in arid regions are not yet fully understood, slowing the progress of research on habitat stress alleviation strategies based on crop physiology.

Previous studies have primarily focused on the effects of single regulatory measures on the physiological traits and yield of spring maize (Zhang et al., 2017b), with relatively few examining the combined effects of multiple regulatory measures on key physiological traits, during critical growth periods and their impact on yield and quality. Therefore, this study hypothesizes that the combined application of magnetoelectric activated water irrigation, plant growth regulators, and root-applied *Bacillus subtilis* can effectively improve photosynthesis and physiological characteristics in crops, thereby enhancing the yield and quality of spring maize in arid regions. Based on this hypothesis, the focus of this study is to explore the impact of magnetoelectric activated water irrigation coupled with amendments on the maize photosynthetic system, and to elucidate the mechanisms by which it promotes grain quality, yield, and yield components. This research aims to provide innovative solutions for efficient maize production in arid regions and offer theoretical support for the sustainable development of modern ecological agriculture.

## 2 Materials and methods

### 2.1 Experimental site conditions

This study was conducted in 2023 at the Xi’an University of Technology experimental station in Gongqingtuan Town, Wensu County, Aksu Prefecture, Xinjiang Uygur Autonomous Region, China (41°27’N, 80°62’E). The station is located at the southern foothills of Tomur Peak in the central Tianshan Mountains, on the northern edge of the Tarim Basin. The experimental area has a typical arid continental climate with distinct seasons, abundant solar and thermal resources, and significant diurnal temperature variation. The average annual temperature is 10.10°C, with an average annual precipitation of about 70 mm. The frost-free period lasts 180 to 220 days, with approximately 3,000 hours of sunshine annually and an annual evaporation rate of about 1,300 mm. Due to the unique geographic environment of Xinjiang, the typical characteristics of oasis agriculture, and the high mineralization of groundwater, secondary salinization frequently occurs in this region, leading to the widespread distribution of saline-alkali land. This study selected the Tailan River irrigation district in Wensu County as the research base, covering an irrigated area of 1.0468 million mu. It is one of the key regions for grain, cotton, oil, and fruit production in Wensu County. **Table 1** presents the physical and chemical properties of the 0-40 cm root zone soil in the experimental area, classified as sandy clay according to international soil texture classification standards, with groundwater depth below 5 m.

**Table 1:** Initial soil physical and chemical properties in the test area.

(cm)	Particle Composition (%)			Bulk Density	Alkali-Hydrolyzable Nitrogen	Available DepthPhosphorus	Available Potassium	Organic Matter	*pH*
	Clay	Silt	Sand	(g/cm^3^)	mg/kg	mg/kg	mg/kg	mg/kg	
0-20	15.89	24.68	59.42	1.56	24.43	12.27	124.50	4.09	8.01
20-40	15.43	25.04	59.54	1.70	12.38	4.57	82.50	2.48	8.15

### 2.2 Experimental materials

In this study, the ‘Xinyu 66’ variety was selected as the research subject, sown on April 28, 2023, and harvested on September 28, 2023, with a total growth period of 153 days. The foliar growth regulator, developed by Xi’an University of Technology, was formulated based on previous studies combining individual growth stimulants. The main ingredients are glycine, proline, *Bacillus subtilis*, fulvic acid, and sodium alginate oligosaccharides in a ratio of 1:1:10:5:1. The chelating agent was combined to prepare the stock solution, which was diluted according to treatment concentration for use. From the seedling stage to the filling stage, it was applied twice each on sunny, windless days, spraying both sides of the maize leaves, for a total of eight applications during the growth period. *Bacillus subtilis* was produced by Junde Ecological Co., Ltd. (Weifang, Shandong Province, China), with a viable count of ≥ 20 billion/g. Before sowing, it was mixed into the 0-40 cm root layer of each experimental plot according to the treatment design.

### 2.3 Experimental design

This experiment was a two-factor study involving a growth regulator (G) and *Bacillus subtilis* (R). All treatments used magnetoelectric activated water and organic fertilizer. To identify the optimal application range of amendments for enhancing photosynthesis and yield of spring maize in arid regions, both G and R were tested at three levels: G was diluted 400 times (G1), 500 times (G2), and 600 times (G3); R was applied at 15 kg/ha (R1), 45 kg/ha (R2), and 75 kg/ha (R3). A control group (CK) was established, which did not apply G and R, and only magnetoelectric activated water irrigation and conventional water and fertilizer management were used. The experiment consisted of 10 treatments, each replicated 3 times in a completely randomized block design, resulting in a total of 30 experimental plots. Each experimental plot measured 8 meters in length and 5 meters in width, covering an area of 40 m².

**Figure 1:**
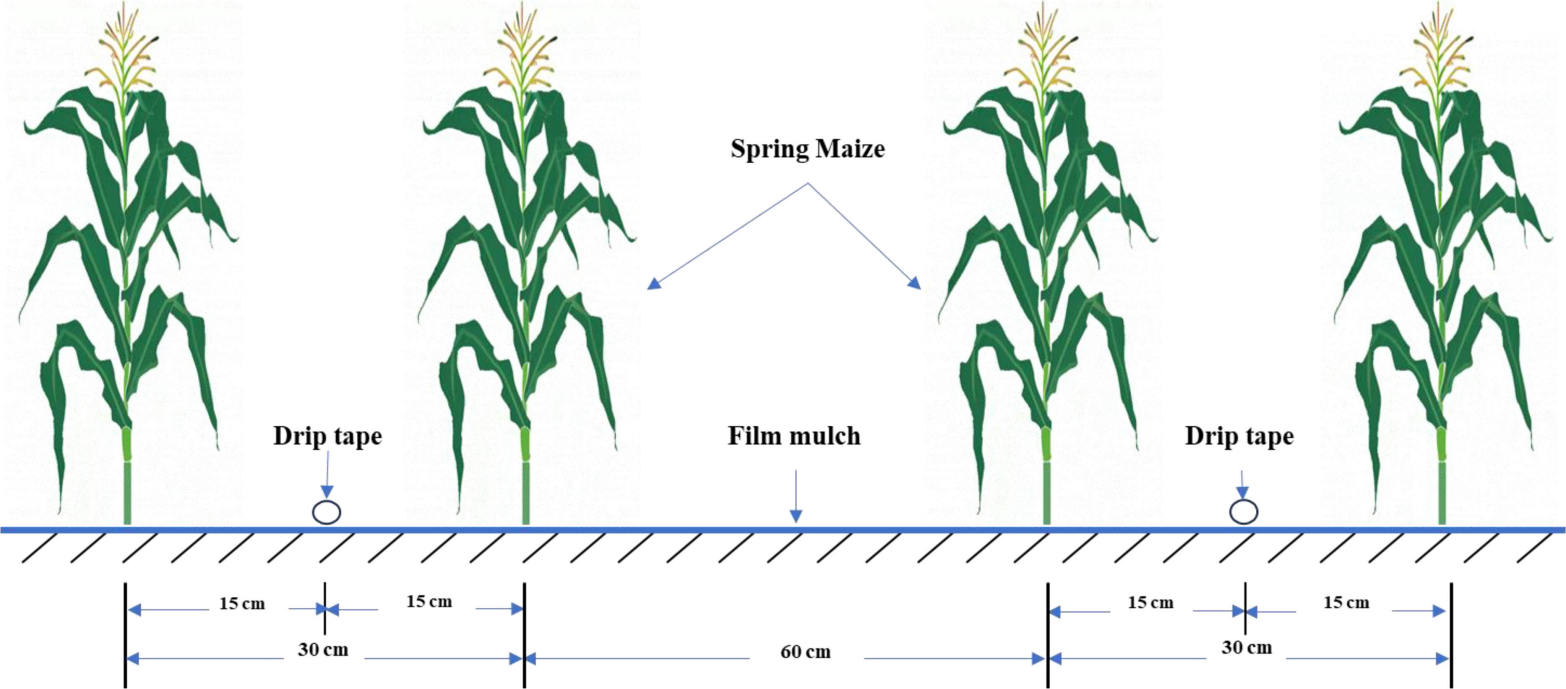
Schematic diagram of spring maize and drip irrigation belt layout.

The planting pattern followed a “one mulching film, two belts, four rows” design, where two drip irrigation belts were laid beneath a single mulching film, with maize seeds sown in four rows, **Figure 1** shows the arrangement of drip irrigation belt and maize. The planting density was 80,040 plants per hectare, with a sowing depth of 5 cm and two seeds per planting hole. The planting density was 82,500 plants per hectare, with a sowing depth of 5 cm and two seeds per planting hole. Before sowing, 60 kg/ha of organic fertilizer and 150 kg/ha of urea were evenly mixed into the 0-40 cm soil layer using a rotary tiller. During the growing period, each treatment received additional applications of urea (46%) at 150 kg/ha, monopotassium phosphate at 150 kg/ha, and potassium sulfate (K_2_SO_4_) at 75 kg/ha. The irrigation amount was controlled at 6.2*10^3^ m³/ha. Drip irrigation was performed using drip belts produced by Xinjiang Tianye Company, with an emitter flow rate of 3.0 L/h and a spacing of 20 cm. The drip belts were laid under the membrane with the seeds during sowing. Fertigation used the 1/4-1/2-1/4 method, where the first 1/4 of the water was clean water, the middle 1/2 included fertilization, and the final 1/4 was clean water again. The irrigation amount for each plot was precisely controlled by a water meter, with a watering cycle of every 7 days.

### 2.4 Measurements and calculations

#### 2.4.1 Meteorological data collection

**Figure 2:**
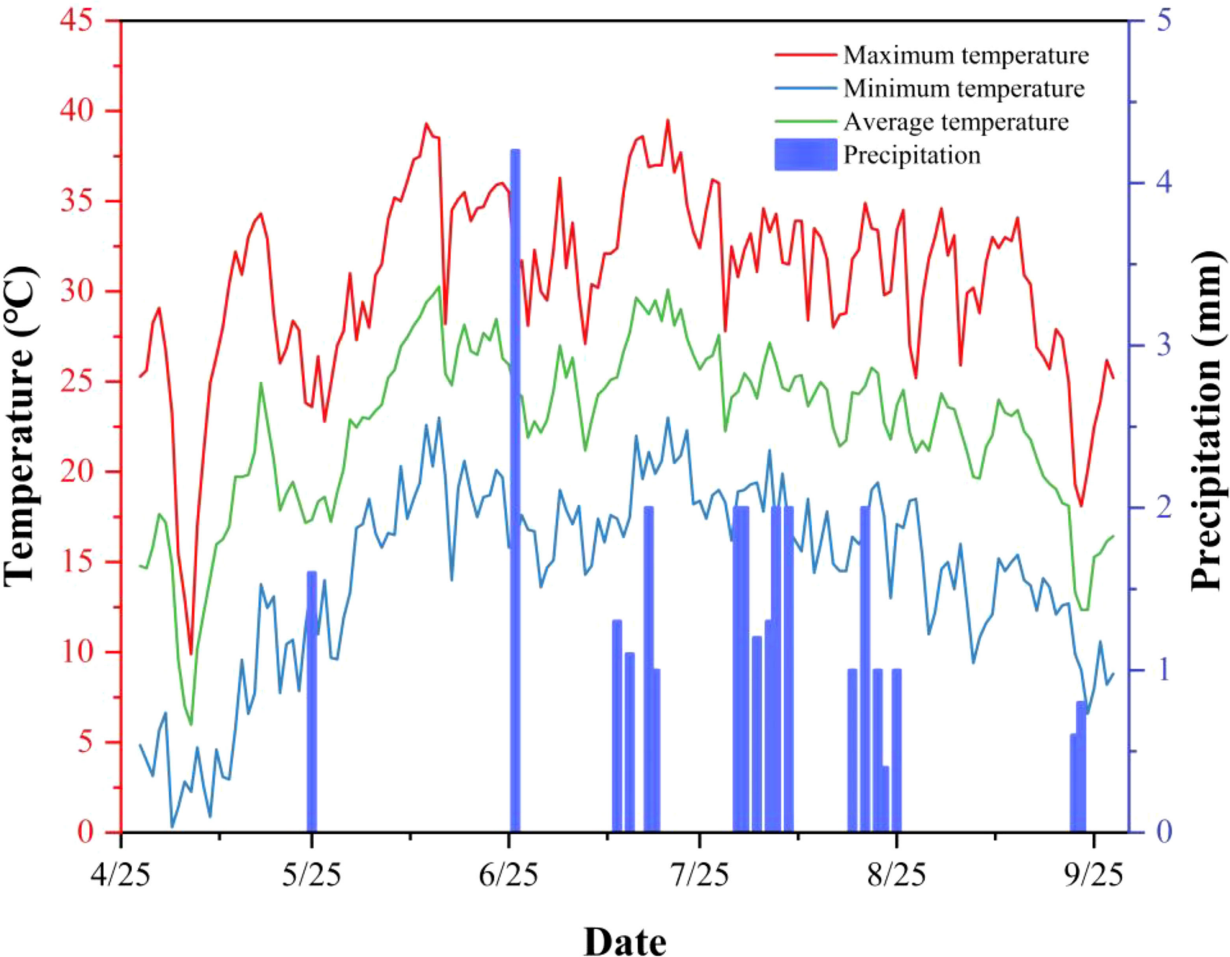
Temperature and rainfall variation throughout the growing season.

The experimental station has an automatic meteorological monitoring station that collects real-time weather data, including temperature, rainfall, wind speed, light radiation, evaporation, relative humidity, and wind direction, throughout each growth stage. Data is automatically recorded every 30 minutes, with the station installed 2 meters above the ground. The variations in meteorological factors during the experiment are shown in **Figure 2**.

#### 2.4.2 Photosynthetic physiology parameter measurement

During the grain-filling period, sunny and windless weather was chosen by checking the weather forecast in advance. The Li-6400xt portable photosynthesis system, produced by PP Systems in the USA, was used to observe three randomly selected uniform and healthy ears of maize per plot between 10:00 and 12:00. Parameters such as net photosynthetic rate (*P_n_*), transpiration rate (*T_r_*), stomatal conductance (*G_s_*), and intercellular CO_2_ concentration (*C_i_*) were measured, along with the light response curve. Each treatment was observed in triplicate, and the average of these observations was used as the treatment value.

#### 2.4.3 Grain nutrient analysis

After harvesting spring maize, the grain samples from each treatment were analyzed for soluble sugar (*SS*, g/kg), soluble protein (*SP*, g/kg), and starch content (*SC*, g/kg). Soluble sugar content was measured using the anthrone colorimetric method, and *SP* content was measured using the Coomassie Brilliant Blue method (Lin et al., 2024a).

#### 2.4.4 Yield and its component analysis

At maturity, the actual yield was measured in each plot. Additionally, 20 ears were randomly selected to determine the 100-kernel weight(*100-kw*)and the number of grains per spike (*Gps*).

#### 2.4.5 Ye Zi-Piao light response model

The Ye Zi-Piao light response model, describing the response of plant leaf photosynthesis to light as proposed by (Ye et al., 2013), is as follows


(1) $$P_{\text{n}}=\alpha_{\text{p}}\frac{1-\beta_{\text{p}}I}{1+\gamma_{\text{p}}I}\text{I}-R_{\text{d}}$$

In the equation, *I* represent photosynthetically active radiation, *α_p_* denotes the initial slope, *β_p_* indicates the light inhibition coefficient, *γ_p_* signifies the light saturation coefficient, and *R_d_* stands for dark respiration rate.

When *R_d_* is assumed to be constant, the *LSP* corresponds to the maximum net photosynthetic rate (*P_nmax_*)​, and is calculated using the following equation:


(2) $$LSP=\frac{\sqrt{\left(\beta_{\text{p}}+\gamma_{\text{p}}\right)/\beta_{\text{p}}}-1}{\gamma_{\text{p}}}$$

The *P_nmax_* is calculated using the following equation:


(3) $$P_{\text{nmax}}=\alpha_{\text{p}}{\left(\frac{\sqrt{\beta_{\text{p}}+\gamma_{\text{p}}}-\sqrt{\beta_{\text{p}}}}{\gamma_{\text{p}}}\right)}^{2}-R_{\text{d}},$$

The light compensation point (*LCP*) can further be calculated using the following equation:


(4) $$\text{L}CP=\frac{\alpha_{p}-\gamma_{p}R_{d}-\sqrt{{\left(\gamma_{p}R_{d}-\alpha_{p}\right)}^{2}-4\alpha_{p}\beta_{p}R_{d}}}{2\alpha_{p}\beta_{p}}$$

#### 2.4.6 Entropy-weighted TOPSIS comprehensive evaluation analysis

The entropy weight method introduces the concept of entropy from information theory, assigns objective weights to each indicator based on the size of its attributes, and calculates the entropy value for each indicator. This approach maximizes the presentation of the original information of the indicators and reduces errors caused by subjective factors. The entropy weight-TOPSIS method, as an effective multi-criteria decision-making approach, facilitates the selection of optimal evaluation schemes. The detailed calculation process is referenced in (Shen et al., 2024).

### 2.5 Data Processing and model validation

#### 2.5.1 Data processing

Data were organized and analyzed using Excel 2021, with variance analysis conducted using SPSS 22.0 for fitting the light response data. Python was used for plotting and model calculations.

#### 2.5.2 Model verification

##### 2.5.2.1 Coefficient of determination

The coefficient of determination (*R^2^*) is a commonly used method for evaluating treatment differences. It measures the extent to which the model explains the variability in the data, with values ranging from 0 to 1, where a value closer to 1 indicates a better model fit. Its calculation expression is as follows:


(5) $$R^{2}=1-\frac{\sum_{i=1}^{n}{\left(O_{i}-S_{i}\right)}^{2}}{\sum_{i=1}^{n}{\left(O_{i}-\bar{O_{i}}\right)}^{2}}$$

Where *O*_i_ is the observed value; *S*_i_ is the model simulation value; $\bar{O_{i}}$ Is the average value of observed values; $\bar{S_{i}}$ Is the average value of the model simulation value; *n* is the number of samples, the same below. *R^2^* can be used as an auxiliary index to evaluate the fitting effect of the model. Still, it should be noted that *R^2^* can only reflect the part of the dependent variable that can be explained by the independent variable and cannot reflect the influence of other influencing factors on the dependent variable. Therefore, it is necessary to combine other evaluation indexes to evaluate the model-fitting effect.

##### 2.5.2.2 Normalized Root Mean Squared Error

*nRMSE* is a commonly used standardized metric for measuring the error between predicted and actual values. It is calculated by dividing the Root Mean Square Error (*RMSE*) by the range or mean of the observed data, making it dimensionless and facilitating comparisons across different datasets. A lower *nRMSE* indicates better predictive performance of the model.

The formula for calculating *RMSE* is as follows:


(6) $$RMSE=\sqrt{\frac{\sum_{i=1}^{n}{\left(\begin{array}{c} Oi-Si \end{array}\right)}^{2}}{n}}$$

Consequently, the formula for calculating *nRMSE* is:


(7) $$nRMSE=\frac{\text{RMSE}}{\bar{O_{i}}}$$

##### 2.5.2.3 Mean Absolute Error

Mean Absolute Error (*MAE*) is a metric used to measure the accuracy of a predictive model by evaluating the magnitude of prediction errors. A smaller *MAE* indicates a more accurate model, while a larger *MAE* signifies poorer prediction accuracy. *MAE* is calculated by summing the absolute differences between predicted and actual values and then dividing by the number of samples. The formula is given by:


(8) $$MAE=\frac{1}{n}\sum_{i=1}^{n}\left|O_{i}-S_{i}\right|$$

## 3 Results and analysis

### 3.1 Characteristics of photosynthetic physiological changes in spring maize

#### 3.1.1 Net photosynthetic rate

**Figure 3:**
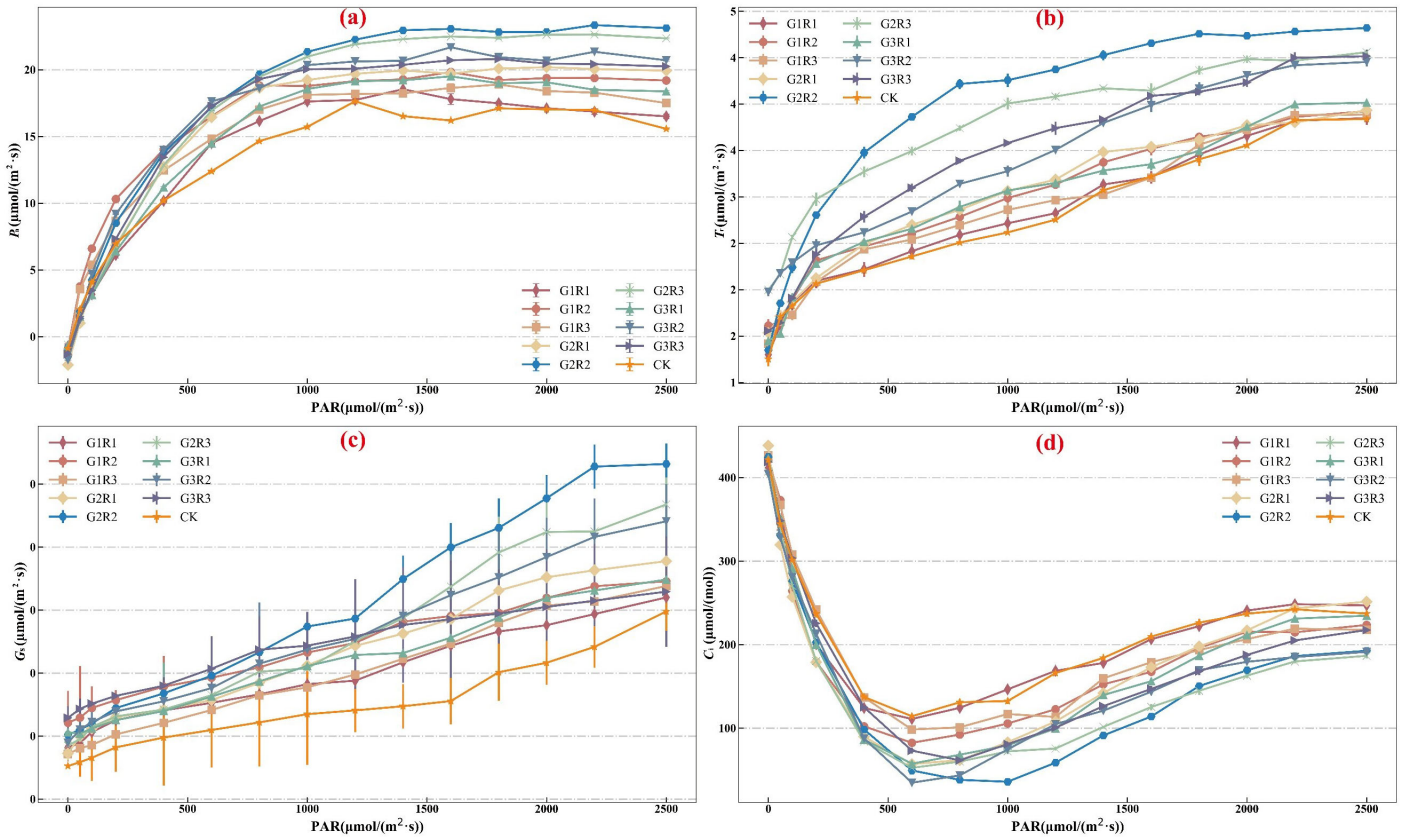
Effects of magnetoelectric activated water irrigation and coupled application of amendments on net photosynthetic rate **(A)**, transpiration rate **(B)**, stomatal conductance **(C)**, and intercellular CO_2_ concentration **(D)** of spring maize.

Studying the light response curve of crops is an important method for understanding photosynthetic characteristics. **Figure 3A** shows the light response curves of spring maize under different coupled treatments of growth regulators. The figure shows that as photosynthetically active radiation (*PAR*) increases, the *P_n_* of spring maize rapidly rises within the range of 0 to 1000 μmol/(m²·s) until it approaches the light saturation point. After reaching the light saturation point, the *P_n_* for each treatment stabilizes and remains constant under strong light conditions. When PAR is 2500 μmol/(m²·s), the G2R2 treatment shows the highest *P_n_* value at 23.13 μmol/(m²·s), followed by G2R3 at 22.36 μmol/(m²·s), and the CK treatment has the lowest *P_n_* value at 17.03 μmol/(m²·s). These results indicate that the coupling of different concentrations of growth regulators significantly promotes the *P_n_* of spring maize. Compared to the control treatment without growth regulators, the *P_n_* value for G2R2 differs by 26.35% from CK, and the differences between treatments are significant (*P<0.05*). The effect of the coupled application of growth regulators on the *P_n_* of spring maize is highly significant. Mean *P_n_* values for spring maize under different light intensities were calculated, showing that the order from highest to lowest *P_n_* value among treatments is G2R2 > G2R3 > G3R2 > G3R3 > G1R2 > G2R1 > G1R3 > G3R1 > G1R1 > CK.

#### 3.1.2 Transpiration rate

**Figure 3B** shows the response of spring maize *T_r_* to the coupled application of growth regulators under different levels of photosynthetically active radiation (*PAR*). The results indicate that at lower light intensities, there is no significant difference in the *T_r_* among the treatments; however, as light intensity increases, the *T_r_* gradually rises. When light intensity reaches 2000 μmol/(m²·s), the rate of increase in transpiration slows down and stabilizes, indicating that spring maize has reached the light saturation point (*LSP*) at excessively high light levels. This suggests that increased light intensity promotes photosynthesis, thereby enhancing transpiration and providing the necessary moisture for photosynthesis. However, excessively high light intensity does not further increase the *T_r_* of the plants. When the light intensity is 2500 μmol/(m²·s), the G2R2 treatment shows the highest *T_r_* at 4.82 μmol/(m²·s), followed by G2R3 at 4.56 μmol/(m²·s), while the CK treatment has the lowest *T_r_* at 3.84 μmol/(m²·s). The *T_r_* values for G2R2 and G2 R3 are 25.58% and 18.77% higher than that of CK, respectively, and the differences between treatments are significant (*P<0.05*). This indicates that the growth regulators effectively increase the *T_r_* of spring maize.

#### 3.1.3 Stomatal conductance

**Figure 3C** illustrates the trend of *G_s_* of spring maize under magnetoelectric activated water irrigation with varying light intensity. The *G_s_* values of all treatments steadily increased with the rise in photosynthetically active radiation (*PAR*), indicating that stomata gradually open in response to light intensity, and photosynthesis intensifies with increasing light intensity. At a light intensity of 2500 μmol/(m²·s), the *G_s_* values for all treatments reached their maximum and showed a trend of further increase. The *G_s_* value under the G2R2 treatment was the highest at 0.27 μmol/(m²·s), followed by G2R3 at 0.23 μmol/(m²·s). Both values were significantly higher than that of CK (0.15 μmol/(m²·s)) (*P<0.05*), with increases of 78.49% and 56.95%, respectively. This indicates that under magnetoelectric activated water irrigation conditions, the coupled application of growth regulators significantly enhanced the stomatal conductance of spring maize.

#### 3.1.4 Intercellular CO_2_ concentration

**Figure 3D** shows the trend of *C_i_* in spring maize under magnetoelectric activated water irrigation as photosynthetically active radiation (*PAR*) varies. As the light intensity increased, *C_i_* decreased rapidly within the range of 0-600 μmol/(m²·s), then gradually and slowly increased, eventually stabilizing. Analyzing different stages, the initial rapid decrease in *C_i_* with increasing light intensity is due to the rapid increase in *P_n_* and the gradual enhancement of photosynthesis. At this stage, the stomata of spring maize are not fully open, so the leaf’s photosynthetic process consumes a large amount of stored CO_2_ to meet the energy required by the increasing light intensity. When *PAR* reached 600 μmol/(m²·s), the average *G_s_* reached 0.08 μmol/(m²·s), and the stomata of spring maize were partially open, allowing the plant to gradually absorb CO_2_ from the environment to continue supporting photosynthesis. At this point, *P_n_* is approaching the light saturation point, and its growth rate gradually slows. Therefore, within the light radiation range of 600-800 μmol/(m²·s), the decreasing trend of *C_i_* in spring maize under the coupled application of growth regulators slows and stabilizes in the range of 34.75-131.03 μmol/mol. As *PAR* continued to increase, *C_i_* gradually rose and stabilized when *PAR* reached 2000 μmol/(m²·s).

### 3.2 Influence of coupling application of amendments on light response curve of spring maize

#### 3.2.1 Model adaptability and fitting analysis

**Figure 4:**
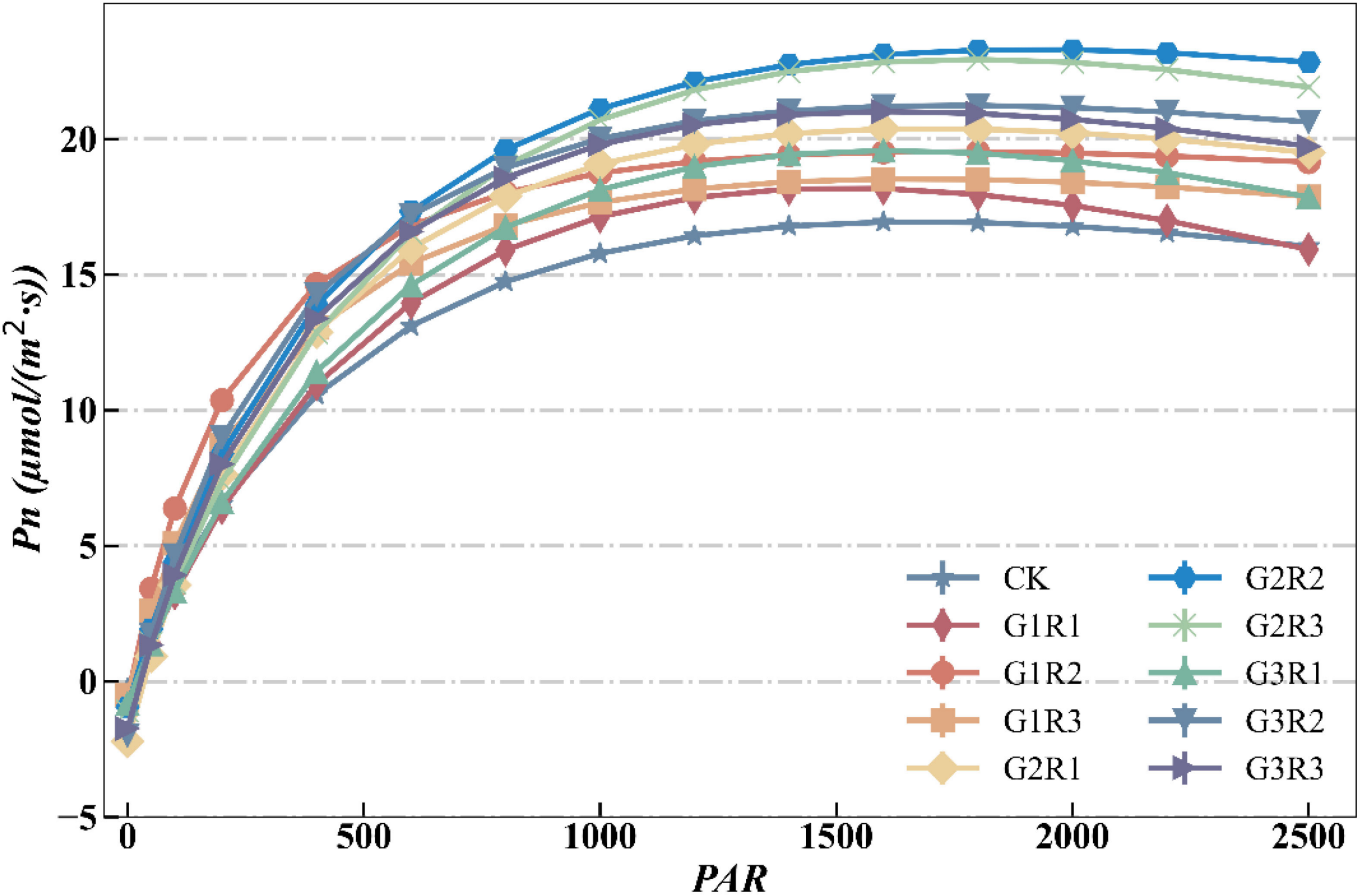
Fitting Of the Ye Zi-Piao light response model under coupled amendment application.

To further investigate the impact of the coupled application of amendments under magnetoelectric activated water irrigation on the photosynthetic characteristics of spring maize, the Ye Zi-Piao light response model was used to fit the field-measured net *P_n_* of spring maize. The fitting results of the Ye Zi-Piao light response model for each treatment are shown in **Figure 4**. As depicted, under magnetoelectric activated water irrigation, the model showed a generally good fit for the net *P_n_* of spring maize.

**Table 2** presents the analysis of the goodness-of-fit for the light response models under the coupling application of magnetoelectric activated water and growth regulators, showing that the Ye Zi-Piao light response model fit well across all treatments. The *R²* and Adj-*R²* values are used to assess the goodness-of-fit of the model, with values closer to 1 indicating a better fit to the data. As shown in **Table 2**, the *R²* and *Adj-R²* values for the different treatments are all close to 1, indicating that the model explains the variance in the target variable well. *MAE* and *nRMSE* are used to evaluate the prediction accuracy of the model. The data show that the *MAE* and *nRMSE* values are small for all treatments, with particularly high prediction accuracy for the G1R1 treatment, where *MAE* is 0.073 and *nRMSE* is 0.004. For all treatments, the *MAE* ranges from 0.073 to 0.434, and the *nRMSE* ranges from 0.004 to 0.028. Overall, the model’s prediction errors are relatively small, and the goodness-of-fit is satisfactory across all treatments. The model demonstrates strong explanatory capability and high prediction accuracy, showing excellent performance in both explanation and prediction.

**Table 2:** Analysis of light response model fit under coupled application of magnetoelectric activated water and amendments.

Treatment	*R^2^*	*Adj-R^2^*	*MAE*	*nRMSE*	Model fitting results
G1R1	0.996	0.995	0.073	0.004	$P_{\text{n}}=0.045\frac{1-0.0001I}{1+0.001I}\text{I}-0.718$
G1R2	0.998	0.997	0.240	0.016	$P_{\text{n}}=0.096\frac{1-0.0001I}{1+0.004I}\text{I}-0.631$
G1R3	0.995	0.993	0.339	0.022	$P_{\text{n}}=0.071\frac{1-0.0001I}{1+0.003I}\text{I}-0.51$
G2R1	0.997	0.996	0.299	0.018	$P_{\text{n}}=0.069\frac{1-0.0001I}{1+0.002I}\text{I}-2.215$
G2R2	0.999	0.999	0.183	0.009	$P_{\text{n}}=0.062\frac{1-0.0001I}{1+0.002I}\text{I}-0.947$
G2R3	0.998	0.997	0.314	0.016	$P_{\text{n}}=0.053\frac{1-0.0001I}{1+0.001I}\text{I}-1.091$
G3R1	0.998	0.998	0.255	0.015	$P_{\text{n}}=0.046\frac{1-0.0002I}{1+0.001I}\text{I}-0.826$
G3R2	0.998	0.998	0.296	0.014	$P_{\text{n}}=0.083\frac{1-0.0001I}{1+0.002I}\text{I}-1.983$
G3R3	0.997	0.996	0.369	0.020	$P_{\text{n}}=0.067\frac{1-0.0001I}{1+0.002I}\text{I}-1.741$
CK	0.992	0.989	0.434	0.028	$P_{\text{n}}=0.045=\frac{1-0.0001I}{1+0.001I}\text{I}-0.301$

#### 3.2.3 Change characteristics of The Ye Zi-Piao light response model characteristic parameter

The light response model reflects the physiological changes of crops under different light conditions and can calculate model parameters such as *P_nmax_*, *LSP*, *LCP*, dark respiration rate (*R_d_*), and light use range (*ΔI*), allowing for a detailed analysis of the crop’s photosynthetic physiological changes. To clarify the impact of coupling growth regulators with magnetoelectric activated water irrigation on the photosynthetic physiological characteristics of spring maize in arid regions, photosynthetic parameters were calculated from the Ye Zi-Piao light response model fitting results, as shown in **Table 3**. The results show that under different coupling treatments of growth regulators, significant differences in photosynthetic parameters of spring maize, such as *α_p_*, *P_nmax_*, *LSP*, *LCP*, *R_d_*, and *ΔI*, were observed. Analysis of **Table 3** reveals that under the coupling application of growth regulators, the effects of *P_nmax_*, *LSP*, *LCP*, and *ΔI* among the Ye Zi-Piao light response model parameters are significant, as detailed below:

**Table 3:** Effect of coupled amendment application on the Ye Zi-Piao light response model fitting parameters.

Treatment		*α_p_*	*β_p_*	*γ_p_*	*R_d_*	*P_nmax_*	*LSP*	*LCP*	*AQY*	*△I*
					µmol/(m^2^·s)	µmol/(m^2^·s)	µmol/(m^2^·s)	µmol/(m^2^·s)		µmol/(m^2^·s)
G1R1		0.04	0.0002	0.001	0.72	18.2	1510.26	16.43	0.03	1493.83
G1R2		0.1	0.0001	0.0036	0.63	19.53	1738.32	6.72	0.06	1731.6
G1R3		0.07	0.0001	0.0025	0.51	18.53	1667.39	7.35	0.04	1660.04
G2R1		0.07	0.0001	0.0019	2.21	20.39	1694.56	34.13	0.05	1660.43
G2R2		0.06	0.0001	0.0015	0.95	23.3	1917.02	15.57	0.05	1901.45
G2R3		0.05	0.0001	0.0011	1.09	22.93	1788.37	21.1	0.03	1767.27
G3R1		0.05	0.0002	0.001	0.83	19.57	1613.88	18.17	0.03	1595.71
G3R2		0.08	0.0001	0.0024	1.98	21.24	1744.09	25.34	0.07	1718.75
G3R3		0.07	0.0001	0.0017	1.74	21.01	1616.32	27.2	0.04	1589.12
CK		0.05	0.0001	0.0014	0.3	16.95	1678.9	6.73	0.04	1672.17
MANOVA	G	**	**	**	**	**	**	**	**	**
	R	**	**	**	**	**	**	**	**	**
	G*R	**	**	**	**	**	**	**	**	**

Different letters in the same column indicate significant differences at the 0.05 level, * indicates significant differences at the 0.05 level, and ** indicates significant differences at the 0.01 level. The same is below.

##### 3.2.3.1 Maximum net photosynthetic rate

At the same growth regulator concentration, the *P_nmax_* of spring maize under various treatments showed a trend of first increasing and then decreasing with the increase in *Bacillus subtilis* dosage. Additionally, under the same *Bacillus subtilis* dosage, the *P_nmax_* of spring maize followed the pattern G2>G3>G1. These results indicate that increasing either the foliar application of the growth regulator or the root application of *Bacillus subtilis* alone significantly enhances the *P_nmax_* of spring maize. Under the condition of coupled application of amendments, the G2R2 treatment had the best effect on increasing the *P_nmax_* of spring maize, reaching 23.3 µmol/(m^2^·s), followed by the G2R3 treatment, which reached 22.93 µmol/(m^2^·s). These were 37.46% and 35.28% higher than the *P_nmax_* of the CK treatment, respectively, with significant differences (*P<0.05*). The G1R1 treatment showed the smallest improvement, at only 18.2 µmol/(m_2_·s), but still increased by 7.37% compared to the CK treatment, with a significant difference (*P<0.05*). The above results indicate that under magnetoelectric activated water irrigation, the coupled application of amendments significantly improves the *P_nmax_* of spring maize in arid regions, but there is an application threshold.

##### 3.2.3.2 Light saturation point and compensation point

The *LSP* is the light intensity at which the net *P_n_* reaches its maximum and does not increase with further light intensity. It can be used to assess the crop’s ability to utilize light. The *LCP* represents a crop’s adaptability to low-light growth environments. The smaller the value, the stronger the crop’s adaptability to low-light conditions, and vice versa. At G1 and G2 plant growth regulators application levels, with the increase in *Bacillus subtilis* dosage, the *LSP* and *LCP* of spring maize under different treatments showed a trend of first decreasing and then increasing. However, at the G3 application level, the *LSP* and *LCP* increased with the increase in R dosage, indicating that within the R2 to R3 range, increasing the dosage can enhance the adaptability of spring maize to low-light environments to varying degrees. Across all plant growth regulators application gradients, the R3 treatment increased by 9.38%, 35.52%, and 7.34% compared to the R2 treatment, with the most significant improvement observed at the G2 gradient, showing significant differences (*P<0.05*).

##### 3.2.3.3 The available range of light intensity

The available range of light intensity (*△I*) reflects the ability of spring maize to utilize different light intensities. Its value is determined by both the *LSP* and the light compensation point *LCP*. Under the coupled application of magnetoelectric activated water irrigation and amendments, when the growth regulator dosage is constant, *△I* increase first and then decrease with the increase in *Bacillus subtilis* dosage. At three growth regulator application levels, the *△I* under R2 dosage was increased by 15.92%, 14.52%, and 7.71% compared to R1, indicating that *Bacillus subtilis* can significantly improve the *△I* of spring maize. However, when the *Bacillus subtilis* dosage is increased from R2 to R3, the *△I* in G1R3, G2R3, and G3R3 treatments decreased by 4.13%, 7.06%, and 7.54% compared to G1R2, G2R2, and G3R2, respectively, indicating that excessive application of *Bacillus subtilis* does not continue to improve the *△I* of spring maize.

### 3.3 Response of grain nutrient changes to amendment application

**Figure 5:**
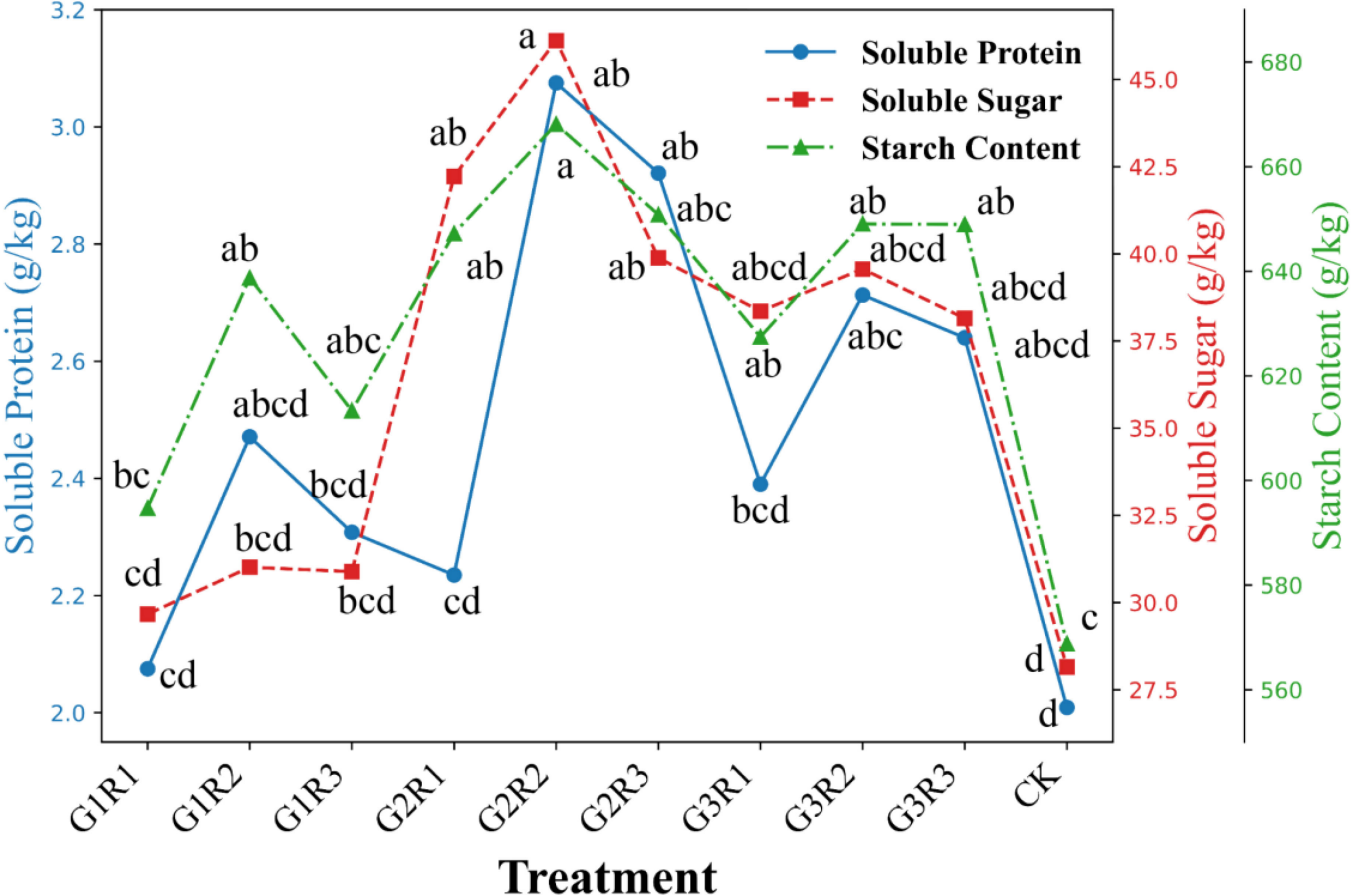
Effect of coupled amendment application on grain nutrients of spring maize under magnetoelectric activated water irrigation.

As shown in **Figure 5**, under magnetoelectric activated water irrigation, the coupled application of amendments increased the *SC*, *SP*, and *SS* in spring maize (*P<0.05*). At the same growth regulator application level, the nutrient content of the grains increased first and then decreased with the increase in *Bacillus subtilis* dosage. In the G2R2 treatment, the *SC*, *SP*, and *SS* contents were 668.11 g/kg, 3.08 g/kg, and 46.12 g/kg, respectively, which were 17.46%, 53.11%, and 63.81% higher than the CK treatment, with significant differences between treatments (*P<0.05*). This indicates that the coupled application of amendments significantly improved the grain nutrition of spring maize. However, at the same growth regulator application level, the effect of *Bacillus subtilis* on improving grain nutrients has an application threshold.

### 3.4 Effect of amendments on the yield and components of spring maize

**Figure 6:**
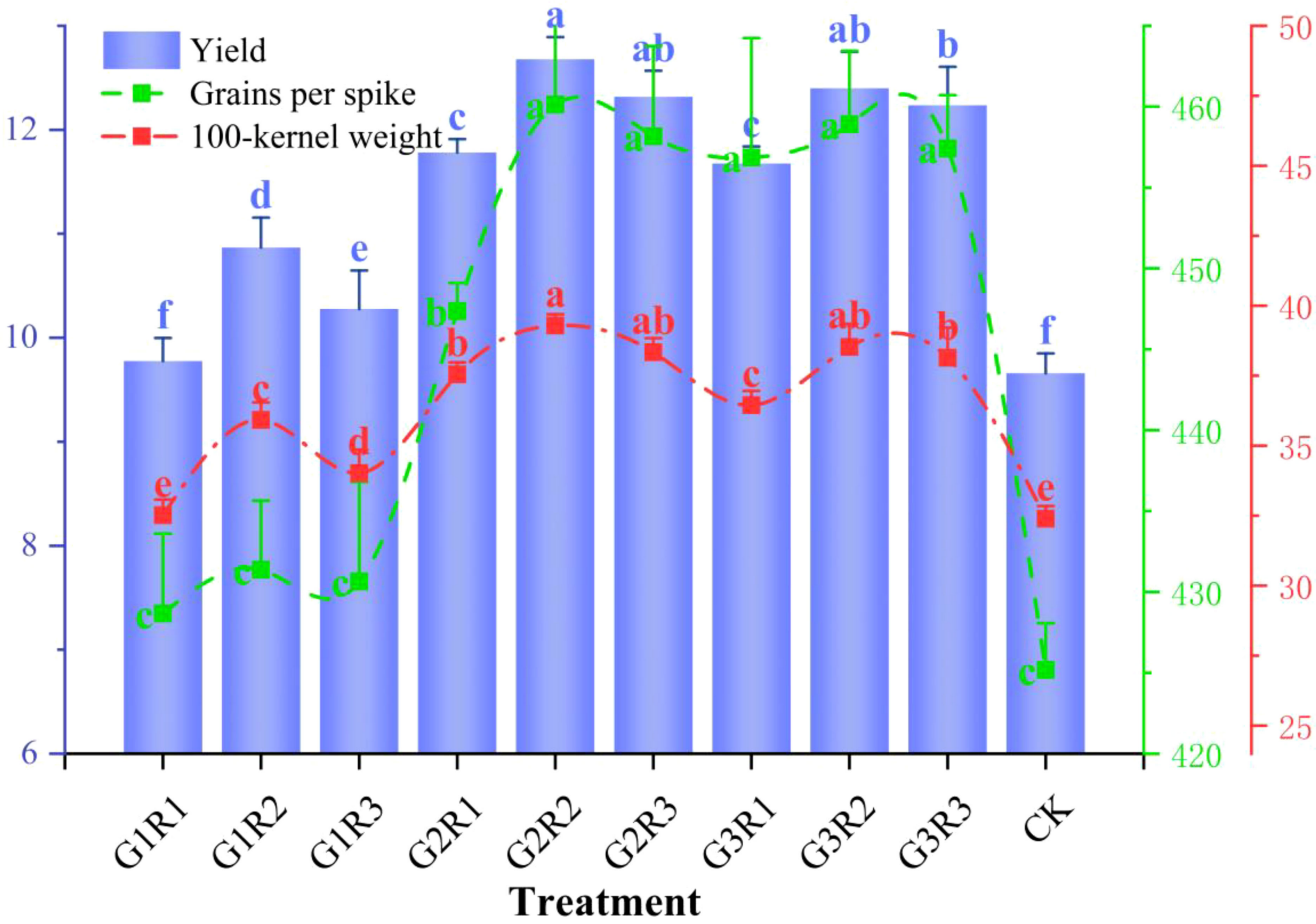
Effect of coupled application of amendments on the yield and components of spring maize. Different letters indicate significant differences at p < 0.05.

**Figure 6** shows the results of spring maize yield and its components under the coupled application of amendments. The treatment effects are ranked as CK < G1R1 < G1R3 < G1R2 < G3R1 < G2R1 < G3R3 < G2R3 < G3R2 < G2R2, indicating that the coupled application of amendments under magnetoelectric activated water irrigation significantly promotes maize yield, with significant differences between treatments (*P<0.05*). The highest yield of 12.68 t/ha was observed in the G2R2 treatment, followed by 12.4 t/ha in the G2R3 treatment, while the lowest yield of 9.66 t/ha was recorded in the CK treatment. The G2R2 treatment increased the yield by 31.27% compared to the CK treatment, with a significant difference (*P<0.05*). The maize yield exhibited a trend of increasing first and then decreasing with the increase in both *Bacillus subtilis* and growth regulator dosages. The *100-kw* and *Gps* are crucial parameters determining the yield level of spring maize. Under the conditions of this study, these parameters showed significant differences in response to amendment application, with trends closely aligned with yield changes. The G2R2 treatment resulted in the highest 100-kernel weight (39.3 g) and grains per spike (460.14 grains), which were 21.33% and 8.22% higher, respectively, than the CK treatment, with significant differences (*P*<0.05).

### 3.5 Correlation analysis of photosynthetic physiological parameters with yield and grain nutrients

**Figure 7:**
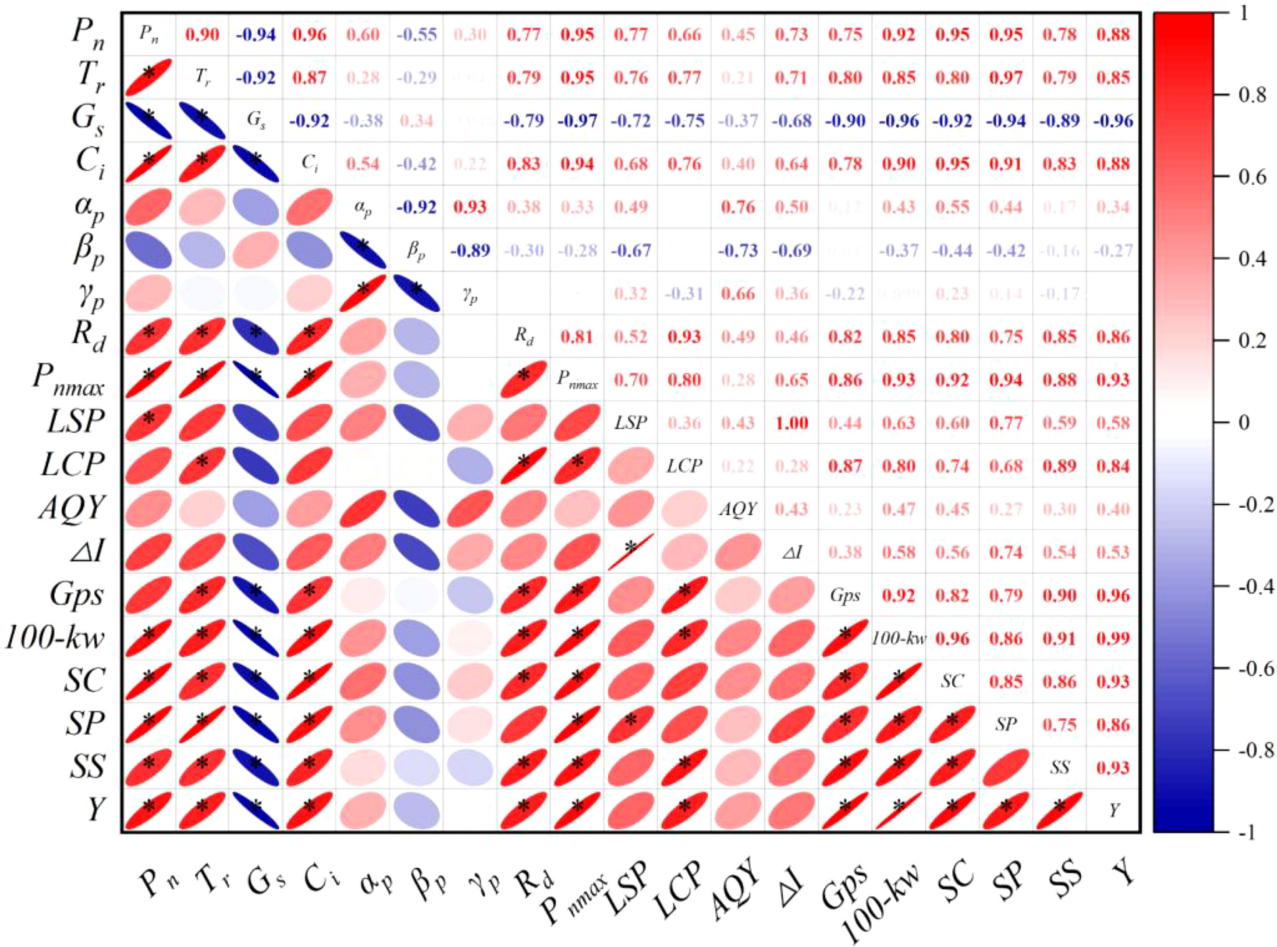
Analysis of the correlation between photosynthetic parameters, light response model characteristics, yield and its components, and grain nutrients.

To clarify the intrinsic relationship between photosynthetic characteristics, yield, and grain nutrients in spring maize, correlation analysis, and Pearson’s correlation coefficient were used to explore the strength of these relationships. **Figure 7** shows the correlation heatmap between parameters. The results indicate a significant correlation between spring maize yield and *LCP* and *R_d_* (*P<0.05*). Additionally, spring maize yield is significantly positively correlated with *100-kw*, *Gps*, *P_nmax_*, *SC*, *SS*, *P_n_*, *SP*, *T_r,_* and *C_i_* (*r≥0.85*). It is significantly negatively correlated with stomatal conductance (*r = -0.96*), with all correlations being significant at *P<0.01*. Spring maize yield is closely related to various physiological and biochemical characteristics, particularly those related to photosynthesis, such as *P_nmax_*. Increases in these indicators are typically associated with higher spring maize yields, and this relationship is quite stable.

The *P_nmax_* is the highest net photosynthetic rate achieved per unit area of plant leaves under optimal environmental conditions. This metric typically reflects the potential and efficiency of plant photosynthesis. Correlation analysis shows that *P_nmax_* has a high correlation with spring maize *Y*, *100-kw*, *Gps*, *SC*, *SS*, and *SP* (*r = 0.86–0.93*), all of which are significant at *P<0.01*.

### 3.6 Comprehensive evaluation of the effects of amendments on photosynthetic characteristics and yield

Through variance analysis of various indicators such as photosynthetic physiological parameters, the Ye Zi-Piao light response model characteristics, and yield in drought-prone spring maize, it was observed that the response to different amendment combinations varied among these indicators. At the same time, a single index does not have the function of comprehensively reflecting the physiological characteristics and yield of spring maize (Li et al., 2019). Comprehensive evaluation metrics were established using photosynthetic characteristics, Ye Zi-Piao light response model parameters, grain nutrient parameters, and yield parameters under different amendment combinations. The weights derived from the entropy method are presented in **Table 4**.

**Table 4:** Weight calculation of indicators using the entropy method.

Dimension	Index	Information entropy value e	Information utility value d	Index Attribute	Index layer weight	Parameter layer weight
Photosynthetic parameter	*P_n_*	0.9983	0.0017	+	0.58%	24.51%
	*C_i_*	0.9337	0.0663	–	22.13%	
	*T_r_*	0.997	0.003	+	1.01%	
	*G_s_*	0.9976	0.0024	+	0.79%	
Ye Zi-Piao light response model characteristic parameters	*γ_p_*	0.9629	0.0371	+	12.39%	71.16%
	*β_p_*	0.9788	0.0212	+	7.07%	
	*α_p_*	0.9846	0.0154	+	5.16%	
	*R_d_*	0.9336	0.0664	+	22.19%	
	*LSP*	0.9991	0.0009	+	0.29%	
	*LCP*	0.9485	0.0515	+	17.20%	
	*AQY*	0.9822	0.0178	+	5.93%	
	*P_nmax_*	0.9981	0.0019	+	0.65%	
	*△I*	0.9992	0.0008	+	0.28%	
Grain nutrient parameters	*SS*	0.9946	0.0054	+	1.82%	3.27%
	*SP*	0.9961	0.0039	+	1.30%	
	*SC*	0.9995	0.0005	+	0.15%	
Yield parameter	*100-kw*	0.999	0.001	+	0.32%	1.05%
	*Gps*	0.9998	0.0002	+	0.07%	
	*Y*	0.998	0.002	+	0.66%	

“+” indicates that the index is a positive indicator, where a larger value represents a better outcome. Conversely, “-” indicates a negative indicator, where a larger value represents a worse outcome.

It can be observed that the total weight of photosynthetic physiological parameters is 24.51%, while the weight coefficient for the Ye Zi-Piao light response model parameters is significantly higher at 71.16% (with the *Rd* parameter having the highest weight coefficient of 22.19%). In comparison, the weight coefficients for grain nutrients and yield are relatively small, at 3.27% and 1.05%, respectively. This indicates that the light response model parameters are crucial for evaluating the effectiveness of amendment combinations.

However, while the entropy weight method only clarifies the weight proportions of each indicator, the TOPSIS method provides results that better align with the actual attributes of the indicators (Tan and Geng, 2020). Therefore, based on the comprehensive evaluation analysis results from the entropy-TOPSIS method (**Table 5**), this study concludes that under the conditions of magnetoelectric activated water irrigation, the combined application of amendments demonstrates the following comprehensive regulatory effects on photosynthetic physiological parameters, the Ye Zi-Piao light response model characteristics, and yield of spring maize in arid regions: G2R2 > G2R3 > G3R2 > G3R3 > G2R1 > G1R2 > G3R1 > G1R3 > G1R1 > CK. The G2R2 treatment has the highest relative closeness value (C = 0.964), indicating the best overall performance and evaluation results.

**Table 5:** Comprehensive evaluation of the effects of combined amendments on photosynthetic physiological parameters, grain nutrient parameters, and yield of spring maize.

Treatment	Positive ideal solution distance D+	Negative ideal solution distance D-	Relative proximity C	Rank
G1R1	3.117	1.737	0.358	7
G1R2	4.346	0.996	0.186	8
G1R3	4.298	0.773	0.152	10
G2R1	1.796	2.919	0.619	4
G2R2	0.526	4.607	0.897	1
G2R3	1.946	2.735	0.584	5
G3R1	2.647	2.042	0.435	6
G3R2	1.363	3.368	0.712	2
G3R3	1.509	3.575	0.703	3
CK	4.397	0.88	0.167	9

## 4 Discussion

As one of the crucial sources of feed and industrial raw materials globally, high-quality maize production holds significant strategic importance for ensuring global food security (Hill et al., 2019). To meet the increasing demand for maize production, enhancing drought resistance and yield in arid regions is a vital strategy. The application of plant growth regulators and Bacillus amyloliquefaciens has played an important role in achieving this goal (Amoanimaa-Dede et al., 2022).

### 4.1 Effects of magnetoelectric activated water irrigation and combined application of amendments on photosynthetic physiological characteristics

Light is one of the main environmental factors regulating plant growth (Tang et al., 2022). Photosynthesis, driven by light, is a crucial chemical process in Earth’s energy cycle and a key source of carbon and energy for crop growth (Zhuang et al., 2024). The efficient functioning of the photosynthetic system is vital for improving crop growth quality (Brestic et al., 2015), increasing yields (Zhang et al., 2020), and maintaining the balance of CO_2_ and oxygen in the ecosystem. Studies have shown that various plant growth regulators and *Bacillus subtilis*, either alone or in combination, significantly enhance crop physiological traits across different regions (Jiang et al., 2023).

This study, under magnetoelectric activated water irrigation conditions, found that the combined application of growth regulators and *Bacillus subtilis* significantly synergized the enhancement of *P_n_*, *T_r_*, and *G_s_* in spring maize leaves, and reduced *C_i_*, thereby significantly improving the photosynthetic capacity of the leaves. The significant effects of these applications may be related to the action of endogenous chemical substances. These endogenous substances are secondary metabolites produced during crop physiological responses (Kazan, 2015). They induce positive responses in crop cells through chemical signals from endogenous plant hormones and metabolic regulation, thereby enhancing stress resistance. Specifically, under the G2R2 treatment, *P_n_*, *T_r_*, and *G_s_* increased by 26.35%, 25.58%, and 78.49% compared to the CK treatment, with significant differences (*P<0.05*). In contrast, the variation in *C_i_* differed from the above parameters. At the same G application rate, *C_i_* initially decreased and then increased with rising R amounts. The CK treatment had a *C_i_* value 37.32% higher than the G2R2 treatment, with significant differences (*P<0.05*). Related research also shows that *Bacillus subtilis* can enhance the health and function of spring maize roots by secreting hormones such as cytokinins (CTK) (Bai et al., 2023). However, there is a reasonable application threshold for this promoting effect. Recent research has found that while the combined application of growth regulators and *Bacillus subtilis* can significantly promote photosynthesis and transpiration, excessive application may lead to resource competition or hormonal imbalance (De Oliveira-Paiva et al., 2024). This may be because high concentrations of G and R could overstimulate plant metabolic activity, accelerating the photosynthesis rate and consuming more CO_2_, thereby reducing *Ci* values. In the experimental design of this study, three different concentrations of growth regulators were selected with the aim of systematically evaluating the effects of these concentrations on maize photosynthetic characteristics, yield, and resource use efficiency. This approach seeks to identify the optimal concentration that can maximize maize photosynthetic assimilation while avoiding resource waste and the risks of excessive application. This gradient design enables a more comprehensive understanding of the effects of growth regulator application, providing scientific support for optimizing maize production. Other studies also indicate that foliar application of growth regulators can regulate crop stomatal opening and closing, optimizing gas exchange and water use efficiency. This effect is particularly important in drought conditions as it can not only improve water use efficiency but also significantly reduce water loss (Amoanimaa-Dede et al., 2022), similar to the conclusions of this study. In this study, at the same concentration of growth regulators, increasing the amount of *Bacillus subtilis* led to a trend where the related parameters first increased and then decreased. This indicates that stomatal opening in spring maize leaves cannot be increased indefinitely by continuously increasing the number of amendments, which is consistent with the conclusions of previous studies. Therefore, in practical agricultural production, it is essential to balance the concentration of combined amendments to achieve the best synergistic effect, which is a key measure for improving crop productivity. Based on this understanding, future research could further explore similar phenomena under different crops and environmental conditions to provide more comprehensive scientific evidence for broader application.

The combined application of amendments not only promotes photosynthesis and transpiration but also significantly affects the light response curve parameters of spring maize. At the same level of growth regulator application, increasing the amount of *Bacillus subtilis* significantly raised *△I*, enhanced antioxidant enzyme activity, and improved the ability of spring maize to utilize light in drought conditions. This effect may be due to *Bacillus subtilis* regulating leaf cell division and elongation, promoting flowering and fruiting, and alleviating plant damage under stress conditions such as drought, high temperatures, and intense light by producing gibberellin (De Oliveira-Paiva et al., 2024). However, further increasing the amount of *Bacillus subtilis* diminished this promoting effect. This may be because excessive application of *Bacillus subtilis* increases endogenous hormones such as ethylene within spring maize plants, and high ethylene concentrations can accelerate the aging of nutritional organs and even leaf senescence, thereby limiting the potential for *Bacillus subtilis* to further enhance the photosynthetic characteristics of spring maize. This phenomenon of promoting effects at low application levels and inhibitory effects at high concentrations is reflected in model parameters such as *P_nmax_*, *LSP*, and *AQY*. *Bacillus subtilis* improves plant growth and development through multiple mechanisms and demonstrates broad application potential in soil improvement and plant nutrition management. Positive feedback from its application in agricultural production has been reported across various crops (Fonseca et al., 2022; Song et al., 2023; Aijaz et al., 2024). Growth regulators significantly promote the photosynthetic physiological characteristics of spring maize through mechanisms such as regulating stomatal opening and closing, increasing chlorophyll content, promoting cell division and elongation, enhancing nutrient absorption, and improving stress resistance. Additionally, growth regulators not only enhance crops’ adaptability to their growing environment but also effectively increase the production of related metabolites (Wang et al., 2024). The combined effects of these mechanisms improve the photosynthetic efficiency of spring maize in arid environments and its adaptability to different light conditions.

### 4.2 Effects of combined application of amendments on yield and composition of spring maize and grain nutrient regulation

In actual production, the yield of spring maize is influenced by environmental factors (such as light, moisture, and temperature), cultivation practices (such as fertilization and irrigation), and varieties. Therefore, it is necessary to conduct specific measurements and analyses for different regions and climatic backgrounds. Achieving high maize yields generally depends on higher*100-kw* and *Gps* (Liao et al., 2024). Improving these two factors is a key approach to achieving high maize yields. Light is a crucial environmental factor determining crop yield, and photosynthetic performance is one of the key factors influencing crop productivity and yield (Khademian et al., 2019). Research indicates that growth regulators show good effects in various crop productions (Kulkarni et al., 2013; Rademacher, 2015). They not only effectively increase yield potential but also improve the physiological metabolism of cells and other nutritional organs by promoting endogenous hormone expression (He et al., 2019), which is consistent with the conclusions of this study. The experimental results show that the G2R2 treatment increased the spring maize yield by 31.27% compared to the CK treatment. Additionally, the combined application of amendments significantly promoted *100-kw* and *Gps*. The G2R2 treatment increased these yield components by 21.3% and 8.22% compared to the non-amendment treatment, with significant differences (*P < 0.05*). Furthermore, the soluble sugar content increased by as much as 63.81%. This indicates that under magnetoelectric activated water irrigation, the combined application of amendments has a significant regulatory effect on yield. Foliar application of growth regulators combined with root application of *Bacillus subtilis* synergistically promoted yield levels of spring maize in drought-affected areas, affecting both above-ground and below-ground parts. The study also found that with increasing amounts of amendments, the yield showed a trend of first increasing and then decreasing. Under the regulation of combined amendment application, the trends in *100-kw* and *Gps* closely matched the trend in yield (**Figure 6**). Therefore, the synergistic effect of foliar application of growth regulators and root application of *Bacillus subtilis* significantly improves maize yield under adverse conditions, under our study conditions, the maximum yield has been increased about 31%, reflect the remarkable application effect in maize production (Xu et al., 2024). Research by (Gao et al., 2022) and others also found that rhizosphere-promoting bacteria can enhance plant growth and alleviate salt stress by secreting auxins, cytokinin, and abscisic acid, significantly improving spring maize’s stress resistance and productivity. This provides new ideas and methods for increasing spring maize yield. Future research should further explore the effects of different types of amendment combinations and their adaptability and stability under various environmental conditions. In-depth research on the complex relationship between rhizosphere-promoting bacteria and environmental factors can improve our understanding of their mechanisms in agricultural production and provide scientific basis for practical applications.

### 4.3 Joint regulation of physiological traits and yield of spring maize in arid areas by amendments

Photosynthesis is the process by which plants convert light energy, carbon dioxide, and water into organic compounds such as *SC*, *SP*, and *SS*. These organic compounds are interconverted within the plant through metabolic networks and various biochemical pathways. The products of photosynthesis form the basis for the synthesis of these compounds, and their accumulation status, in turn, affects the efficiency of photosynthesis and the overall growth state of the plant. Maize plants with high photosynthetic efficiency generally produce more soluble sugars (Zhang et al., 2020). Our study results show that *P_n_*, *P_nmax_*, and *SS* have very significant correlations (0.78 and 0.88, *P<0.01*). These sugars act as energy reserves and transport substances within the plant, positively influencing the growth, development, and metabolic activities of spring maize. These sugars can also be further converted into starch (Begam et al., 2024). Maize plants with higher photosynthetic efficiency synthesize and accumulate greater amounts of starch during the day. This starch, as an energy reserve, is broken down into sugars at night or when needed, providing ample energy for subsequent metabolic processes (Qiao et al., 2022). In this study, under the combined application of the amendments, the correlations between *P_n_*, *P_nmax_*, and *SC* were 0.95 and 0.92 (*P<0.01*), respectively, demonstrating a strong association between *P_n_* and *SC*, consistent with the aforementioned conclusions. Additionally, the correlations between *SC*, *SP*, *SS*, and *Y* were 0.93, 0.86, and 0.93, respectively, all showing very significant relationships (*P<0.01*), further confirming the previous research conclusions. These findings indicate that the combined application of amendments can enhance grain nutrition by regulating photosynthetic characteristics. Additionally, the interactions of these physiological and biochemical traits determine the photosynthetic physiological characteristics and yield performance of maize.

Multi-Criteria Decision-Making (MCDM) analysis methods provide researchers facing complex multi-scenario optimization problems with a powerful tool (Wang et al., 2019a)and are favored by many scholars for their objective results. However, for the same analysis object, different MCDM methods can yield significantly different results due to variations in their calculation processes. Therefore, choosing the most appropriate MCDM method is crucial for making accurate and effective decisions. TOPSIS evaluation analysis is a multi-criteria decision-making method that ranks and selects evaluation results by analyzing the degree of closeness between the evaluation scheme and the ideal solution (Çelikbilek and Tüysüz, 2020). Due to its simplicity, ease of use, and wide applicability, it has been successfully used in multi-scenario analysis and optimization problems, making the entropy weight-TOPSIS method widely applied in multi-criteria comprehensive research (Chen, 2020). This study uses the entropy method to determine the weights of key indicators such as photosynthetic parameters, light response model characteristics, grain nutrient yield, and its components and then applies the TOPSIS method for quantitative evaluation and analysis. This approach provides data support for qualitative analysis and a theoretical basis for developing effective production measures for spring maize in arid regions. The results of this study show that the weights of the photosynthetic parameters and Ye Zi-Piao light response model characteristic parameters account for 95.67%, making them crucial factors in determining the yield of spring maize. The entropy-weight TOPSIS method identified that under magnetoelectric activated water irrigation, the optimal response combination for the application of amendments is the G2R2 treatment. This combination not only enhances the photosynthetic efficiency of spring maize in arid regions but also works synergistically to improve both the yield and grain quality. It provides strong scientific evidence and technical support for addressing agricultural production challenges in these areas.

**Figure 8:**
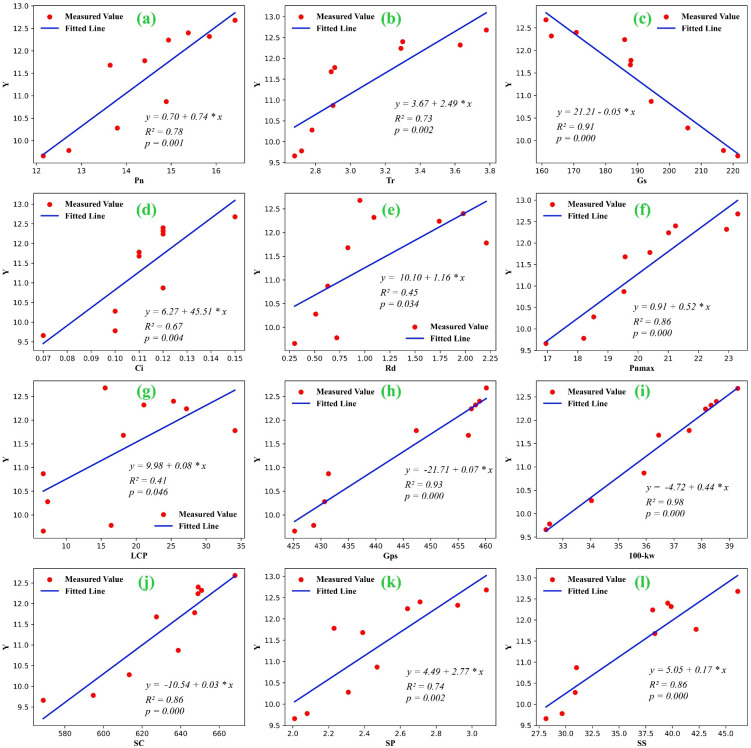
Regression analysis of spring maize yield and net photosynthetic rate **(A)**, transpiration rate **(B)**, stomatal conductance **(C)**, intercellular CO_2_ concentration **(D)**, dark respiration rate **(E)**, maximum net photosynthetic rate **(F)**, light compensation point **(G)**, number of grains per spike **(H)**, weight of 100 grains **(I)**, starch content **(J)**, soluble protein **(K)** and soluble sugar **(L)**.

Building on the correlation analysis, an in-depth analysis of the linear regression relationships between photosynthetic parameters, yield and its components (*100-kw* and *Gps*), light response model parameters (*R_d_*, *P_nmax_*, and *LSP*), and grain nutrients (*SC*, *SP*, and *SS*) was conducted. **Figure 8** shows significant linear correlations between these parameters, with the regression model’s coefficient of determination (*R²*) ranging from 0.45 to 0.98. All models reached highly significant levels (*P<0.01*), indicating that the models have strong explanatory power and effectively reflect the quantitative relationships between spring maize yield, photosynthetic parameters, model characteristic parameters, yield components, and grain nutrients (Ma et al., 2022). Therefore, in arid regions, amendments can significantly enhance grain nutrient quality and increase yield, making it an effective approach to boosting spring maize production.

In summary, arid regions face environmental pressures such as water scarcity and high evaporation, which pose significant challenges to crop production (Akumaga et al., 2023). The combined application of soil amendments effectively enhances crop resistance to these environmental pressures, synergistically promoting photosynthesis and nutrient absorption, thereby significantly increasing the yield and quality of spring maize in arid regions (Rawat et al., 2023). Future research could further explore the effects of different application timings, frequencies, and crops to optimize amendment usage strategies and enhance their effectiveness.

## 5 Conclusion

Through the study of the combined application of growth regulators and *Bacillus subtilis* under magnetoelectric activated water irrigation on the photosynthetic physiological traits, grain nutrients, yield, and composition of spring maize in arid regions, the following conclusions are drawn:

1. Under the condition of combining magnetoelectric activated water with growth regulators and *Bacillus subtilis* application, the G2R2 treatment effectively increased the *P_n_* (26.35%), *T_r_* (25.58%), and *G_s_* (78.49%) of spring maize compared to the CK treatment, while reducing *C_i_* by 37.32%. The research design significantly synergistically enhances the photosynthetic characteristics of spring maize. There is a high correlation between yield and photosynthetic parameters (*r* > 0.85), and the linear regression model demonstrates a high level of stability (*R²* > 0.67). Improving photosynthetic performance is an effective way to increase the yield of spring maize. The combined application of growth regulators and *Bacillus subtilis* has a significant effect, with a reasonable application threshold. Excessive application may lead to resource competition or hormone imbalance. In practical production, the application rates of growth regulators and *Bacillus subtilis* should be optimized and regulated based on the specific needs of the crop to achieve optimal growth and yield improvement.
2. The entropy weight-TOPSIS multi-criteria comprehensive evaluation method fully reveals the response of photosynthetic physiological characteristics, and yield of spring maize to the combined application of amendments in arid regions. Under the G2R2 treatment, the yield reached 12.68 t/ha, with a 31.27% increase. Meanwhile, this treatment significantly improved the *100-kw*, *Gps*, and *SS*, with increases of 21.3%, 8.22%, and 63.81%, respectively, compared to the CK treatment. This combination benefits yield enhancement and quality improvement of spring maize in arid regions. Under magnetoelectric activated water irrigation conditions, our research suggests that the treatment with 500 times diluted growth regulator (G2) combined with 45 kg/ha (R2) of *Bacillus subtilis* is an appropriate agent combination for the arid regions of southern Xinjiang, China.

In conclusion, this study highlights the key factors and application strategies for improving photosynthetic efficiency, yield, and grain quality of spring maize in arid regions, providing theoretical guidance and technical support for agricultural decision-makers in these areas.

## Data Availability

The original contributions presented in the study are included in the article/supplementary material. Further inquiries can be directed to the corresponding author.
